# Archaeal Diversity in Biofilm Technologies Applied to Treat Urban and Industrial Wastewater: Recent Advances and Future Prospects

**DOI:** 10.3390/ijms140918572

**Published:** 2013-09-09

**Authors:** Kadiya Calderón, Alejandro González-Martínez, Cinta Gómez-Silván, Francisco Osorio, Belén Rodelas, Jesús González-López

**Affiliations:** 1 Environmental Microbiology Group, Department of Microbiology, Faculty of Pharmacy, and Institute of Water Research, University of Granada, Campus de Cartuja s/n, Granada 18071, Spain; E-Mails: cintagomez@ugr.es (C.G.-S.); mrodelas@ugr.es (B.R.); jgl@ugr.es (J.G.-L.); 2 Environmental Microbiology Group, Department of Civil Engineering, and Institute of Water Research, University of Granada; Campus de Cartuja s/n, Granada 18071, Spain; E-Mails: agon@ugr.es (A.G.-M.); fosorio@ugr.es (F.O.)

**Keywords:** Archaea, biofilm, biofouling, wastewater treatment, WWT, membrane bioreactor, MBR, granular sludge

## Abstract

Biological wastewater treatment (WWT) frequently relies on biofilms for the removal of anthropogenic contaminants. The use of inert carrier materials to support biofilm development is often required, although under certain operating conditions microorganisms yield structures called granules, dense aggregates of self-immobilized cells with the characteristics of biofilms maintained in suspension. Molecular techniques have been successfully applied in recent years to identify the prokaryotic communities inhabiting biofilms in WWT plants. Although methanogenic Archaea are widely acknowledged as key players for the degradation of organic matter in anaerobic bioreactors, other biotechnological functions fulfilled by Archaea are less explored, and research on their significance and potential for WWT is largely needed. In addition, the occurrence of biofilms in WWT plants can sometimes be a source of operational problems. This is the case for membrane bioreactors (MBR), an advanced technology that combines conventional biological treatment with membrane filtration, which is strongly limited by biofouling, defined as the undesirable accumulation of microbial biofilms and other materials on membrane surfaces. The prevalence and spatial distribution of archaeal communities in biofilm-based WWT as well as their role in biofouling are reviewed here, in order to illustrate the significance of this prokaryotic cellular lineage in engineered environments devoted to WWT.

## 1. Archaea and Biofilms: An Introduction

Archaea is one of the three domains of life distinguished by Carl Woese by phylogenetic analysis based on 16S rRNA genes [1]. They are abundant and metabolically-diverse microorganisms which coexist with Bacteria and Eukarya in most Earth environments; however, they remain the least well known of the branches of the phylogenetic tree of life, despite the many efforts made to investigate their role in natural and engineered systems [2]. Their diversity remains rather unexplored, although it has been estimated to be comparable to that observed for Bacteria [2]. The physiological functions of the Archaea identified in mixed microbial communities suggest their significant role in the biogeochemical cycles of the planet, maintaining the flow and recycling of the nutrients in many environments [3]. In particular, the recent discovery of ammonia-oxidizing Archaea (AOA) changed the classical view of the nitrogen (N) cycle, as AOA are currently regarded as the main ammonia-oxidizing organisms in oceans and geothermal habitats [4].

Biofilms are complex, spatially-structured multicellular communities, formed on the surfaces or interfaces of materials of both organic and inorganic nature [5]. Biofilms are known to have existed since the discovery of microorganisms, as they were first visualized by van Leewenhoeck in the XVII century [6], and have become accepted as the dominant microbial life style in nature. Cell aggregation and surface adhesion provide a protected mode of growth, enabling survival in hostile environments. The nature of biofilm structure is dynamic, as the cells anchored to the surface eventually disperse and revert into the planktonic mode of living, which then enables the colonization of new niches [7].

The steps that lead to the formation of microbial biofilms have been extensively described by different authors [5,8,9]. It is generally accepted that the process starts when microbes associated with a surface change from a reversible to an irreversible mode of attachment to it, followed by the aggregation of cells and their subsequent proliferation. The cells in the biofilm are encased in a matrix of self-produced polymers of heterogeneous nature (lipids, polysaccharides, extracellular nucleic acids or proteins), referred to as the EPS (extracellular polymeric substances), which fulfill important functions [5]. The extraordinary tolerance of biofilms to antimicrobial compounds, heavy metals and other damaging agents derives from a complex mixture of physical, chemical and physiological factors: the metabolic heterogeneity of the community, the particular physiological state of the microorganisms in the different biofilm layers, the support of syntrophic and other mutualistic interactions, and the development of specialized subpopulations of resistant phenotypes and persister cells [10]. The relative contribution of each of these mechanisms (and possibly others) varies with the type of biofilm and the nature of the environment where they develop [3].

Since biofilms have been recorded in fossils more three billion years old, this lifestyle is acknowledged as an ancient feature of prokaryotes [7]. In past decades, the focus of researchers was centered mainly on bacterial biofilms; however, thanks to recent advances in monospecies cultures, it has been possible to demonstrate that Archaea are also capable of attaching to biotic and abiotic surfaces and developing biofilms [3]. Biofilm formation in the environment by members of the archaeal Phyla *Euryarchaeaota*, *Crenarchaeota*, *Korarchaeota* and *Thaumarchaeota* is well documented, particularly in extreme habitats [11–14]. It is also well known that Archaea are present in biofilms of engineered habitats, such as acid-mine drainages, aquarium biofilters, or wastewater treatment (WWT) plants based on different technologies [14–19]. This review aims to summarize the current knowledge on the characteristics of Archaea and the roles they play under the biofilm lifestyle in WWT systems, with particular emphasis on their occurrence, diversity and attributed functions.

## 2. Biofilm Systems Associated to WWT

Biological WWT technologies based on the use of biofilms are broadly applied for the removal of organic matter, nitrogen and other anthropogenic contaminants occurring in wastewater. Mixed-population biofilms develop in these systems, normally requiring the addition of carrier inert materials to provide a supporting surface. Although many configurations of biofilm-based WWT plants have been devised, they can be classified into two broad types: fixed-bed reactors, which regard all systems in which the biofilms develop on a static media; and expanded-bed reactors, which include carrier media subjected to continuous motion driven by stirring or an air flux [20]. Amongst other advantages compared to the more generally applied conventional activated sludge (CAS) technology, biofilm-based systems are simple to control and maintain, reduce space needs, lower cost, and minimize unwanted odors and noise [21].

**Figure 1:**
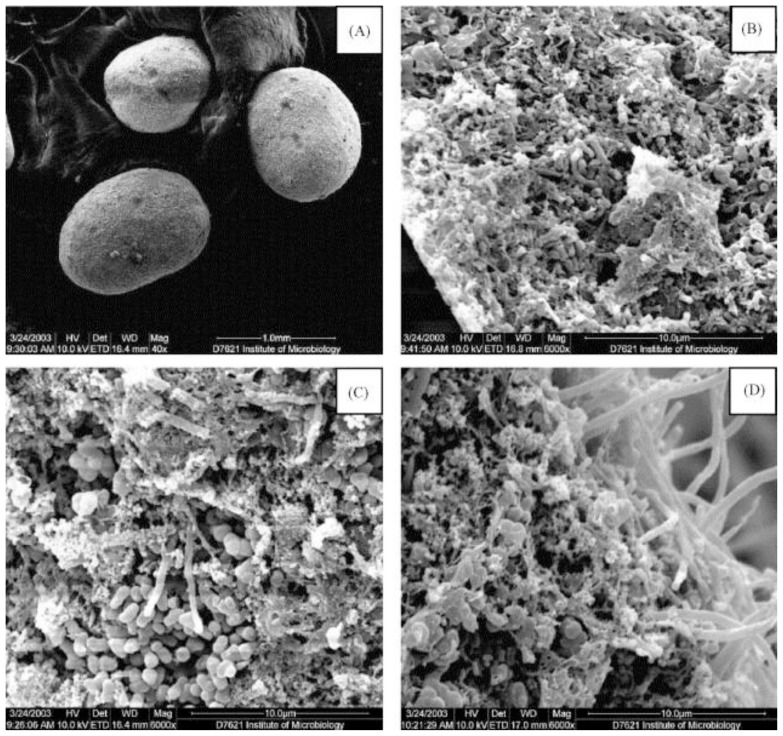
Scanning electron micrographs of anaerobic granular sludge cultivated in an Expanded Granular Sludge Bed (EGSB) reactor. (**A**) Morphology of anaerobic granules used (40× magnification); (**B**,**C**,**D**) Inner structure of anaerobic granules (6000× magnification). Reprinted from [23], Process Biochemistry, Vol. 40, Wang, J. and Kang, J., The characteristics of anaerobic ammonium oxidation (ANAMMOX) by granular sludge from an EGSB reactor, Pages 1973–1978, Copyright (2005), with permission from Elsevier.

Different WWT systems use granular sludge (GS), based on the aggregation of microbial biomass in structures named granules, which are regarded as suspended biofilm systems and dissimilar to flocs in their shape, structure and substrate diffusion properties [22,23]. Their typical morphology and inner structure is shown in Figure 1 [23]. Granules develop in the absence of a supporting surface by the auto-immobilization of the microorganisms, and are functionally described as concentric layers of densely-packed, near-spherical biofilms, each of which is inhabited by different microbial trophic groups [24,25]. Each granule is a functional unit in itself, comprising all the different microorganisms necessary for the degradation of wastewater, producing biomass and EPS [26].

GS has advantages over the conventional floc aggregates and biofilms developed on supporting media, with the main one being the wider surface area provided for the biofilm [27]. GS can develop in both aerobic and anaerobic systems, provided that certain conditions are given in bioreactor design [28]. The granulation process and granule stability are affected by many operating and external factors such as temperature, hydraulic retention time (HRT), organic loading rate (OLR), nutrient availability, and the presence of divalent cations and heavy metals [25,26,28,29]. In this review, only factors known to have an influence on archaeal diversity will be discussed.

## 3. Archaeal Communities in Anaerobic Bioreactors

Anaerobic bioreactors are used for the degradation of organic matter, generating methane as a value-added by-product [30]. The methanogenic metabolism is an exclusive feature of a group of prokaryotes classified in the Phylum *Euryarchaeota*, which is currently divided into six orders: *Methanobacteriales*, *Methanococcales*, *Methanomicrobiales*, *Methanosarcinales*, *Methanopyrales* and *Methanocellales* [31,32]. There are also a number of 16S rRNA gene types that are often retrieved from WWT which presumably belong to as yet uncultivated archaeal taxa with metabolic functions close to those of known methanogens [33]. This is the case of sequences assigned to the WSA2 (or ArcI) group, which is considered to be an archaeal taxon at the class level [34].

Despite their ample phylogenetic, morphological and physiological diversity, methanogens only use a limited number of substrates to obtain energy. Most methanogens are restricted to using H_2_ + CO_2_ or formate [31]. Some members of the *Methanomicrobiales* use secondary alcohols, and *Methanosarcinales* are the more metabolically versatile, being often able to use methyl group-containing compounds and also comprising the only acetoclastic methanogens, *Methanosarcina* spp. and *Methanosaeta* spp. [30,31].

Studies on the microbial diversity of anaerobic bioreactors have increased in the last 20 years, fuelled by the introduction of molecular cultivation independent methods. A wide array of primers and probes targeting phylogenetic markers of methanogens are currently available [34]. The archaeal diversity in this type of systems is limited when compared with bacterial diversity. The sequences retrieved from anaerobic reactors belong mostly to members of the *Euryarchaeota* phylum, although the occurrence of crenarchaeotal sequences has also been reported [33,35–37]. Despite the many differences in wastewater nature, bioreactor design and operating conditions, an overall conclusion is that the dominant Archaea are the methanogens, which usually belong to the *Methanobacteriaceae*, *Methanosarcinaceae* and *Methanosaetaceae* [35,37–43]. In most of the systems studied, both acetoclastic and hydrogenotrophic CO_2_-utilizing methanogenic Archaea coexist. It has been suggested that this configures the minimal archaeal microbiota required for stable anaerobic digestion [35].

**Figure 2:**
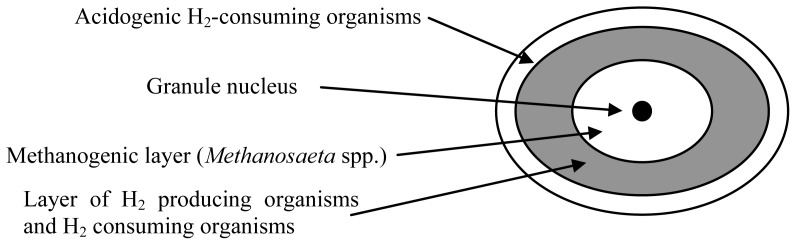
Anaerobic granule formation, according to the model of McHugh *et al*. [50].

Granular biomass formation by archaeal populations has been widely studied in anaerobic digestion processes [22,35,37–41,44–49]. From the technological point of view, these systems comprise mainly the UASB and expanded granular sludge bed (EGSB) reactors [28]. In 2003, McHugh *et al*. [50] proposed a layered structure for the anaerobic granules in which a central core of acetoclastic methanogens is surrounded by a layer of hydrogen- or formate-producing acetogens and hydrogen- or formate-consuming methanogens. The proposed granule structure provides an outside layer of microorganisms that hydrolyze and acidify complex organic matter [51] (Figure 2). Once the reactors have been seeded with anaerobic sludge and wastewater, the wastewater flows in the upward direction through the sludge and granule formation slowly occurs spontaneously under appropriate conditions of substrate and nutrient availability, pH, alkalinity, and upflow velocity [26].

Several other types of anaerobic reactors have been successfully designed and applied to a lesser extent for the treatment of a wide range of organic-rich wastewaters (reviewed by Tabatabaei *et al*. [41]). Anaerobic reactors comprising a fixed-bed or an expanded-bed phase for biofilm development have been widely evaluated for the treatment of urban and industrial wastewaters [35]. The bioreactors can be entirely designed as biofilm-based, or include a biofilm phase associated to a granular phase as part of an UASB or an anaerobic baffled reactor (ABR). Methanogenic Archaea adhere preferentially to packing support materials [52]; thus, the performance of methanogenesis is higher in bioreactors when such a surface is provided for biofilm development [42]. Besides, a biofilm phase helps to improve and maintain granulation in the associated granular phase [53]. Several packing materials, such as charcoal, gravel, brick pieces, pumice stones, coconut coir, carbon fiber, nylon fiber and plastic pieces have been tested [54,55].

Some efforts have been made to describe the core prokaryotic microorganisms essential for the anaerobic degradation of organic matter, providing evidence that the archaeal communities are indeed composed of a restricted number of operational taxonomic units (OTUs). Leclerc *et al*. [35] used molecular methods (16S rRNA-based single-strand conformation polymorphism fingerprints and clone libraries) to analyze and compare the diversity of Archaea in 44 anaerobic bioreactors based on different technologies and treating diverse types of wastes. Most frequently, a combination of sequences phylogenetically close to *Methanobacterium* spp. and *Methanosaeta concilii* was found. The authors also concluded that the distribution of the archaeal species was not strongly influenced by the nature of the wastewater, but depended in part on the type of bioreactor technology. The stirred-tank digesters were able to support a community of a higher diversity compared to the biofilm-based technologies. Some archaeal populations were often found to exclude each other, showing preference for a particular type of bioreactor design. For instance, *Methanosarcina frisus* was prevalent in stirred-tank and fixed-film digesters, but occurred in low levels in upflow anaerobic sludge bed (UASB) reactors, which in contrast favored the presence of *Methanosaeta* spp. Rivière *et al*. [33] compared seven mesophilic (29–37 °C) digesters used for sludge reduction in urban WWT plants across France, Germany and Chile, by analyzing large clone libraries of archaeal 16S rRNA gene fragments. In total, 69 different archaeal OTUs were found, with the majority of the sequences (62.4%) being affiliated to three OTUs shared among 4–7 of the analyzed digesters, and 24 other OTUs (34%) shared by 2–4 digesters. The last 42 OTUs were specific for one digester (3.6% of the sequences). In agreement with the study by Leclerc *et al*. [35], most of the recognized OTUs were affiliated to methanogenic Archaea (*Methanosarcinales*, *Methanomicrobiales*, *Methanobacteriales* and ArcI group) with *Methanosaeta* spp. as the main acetoclastic methanogen. Interestingly, the most represented OTU belonged to the ArcI lineage, and members of ArcI were the dominant archaeal populations (41%–69% of the sequences) in four of the analyzed anaerobic digesters.

The structure of archaeal communities in fixed-bed or expanded-bed biofilm systems has been scarcely investigated. In the study by Leclerc *et al*. [35], one fixed-bed reactor and seven fluidized-bed reactors treating diverse types of industrial wastewaters (brewery, winery, dairy) were evaluated, finding *Methanobacterium*, *Methanosaeta* and *Methanosarcina* as the prevalent genera. Comparing the results with those of GS systems, the authors concluded that all the fixed-bed and fluidized-bed reactors exhibited similar and distinctive archaeal diversity patterns, suggesting that the required attachment of cells to the supporting media strongly conditioned community structure. More recent studies indicate that *Methanobacteriales* and *Methanomicrobiales* coexist in fixed-film anaerobic reactors. Zhang *et al*. [42] explored the community dynamics in different compartments of two mesophilic fixed-bed anaerobic baffled reactors (FABRs) by the generation of archaeal clone libraries of the 16S rRNA gene and quantitative real-time PCR (qPCR). Although *Methanobacteriales* and *Methanosaeta* dominated the seed sludge used to inoculate the FABRs, *Methanomicrobiales* increased 30- to 42-fold after 32 days of operation. *Methanolinea* and *Methanospirillum* showed a preference to colonize the carbon fiber support during the start-up period, particularly in the last compartment of the system, where methanogenesis took place at the highest rate. Rademacher *et al*. [43] characterized the community structure of microbial biofilms developed in a thermophilic biogas system, by means of massive parallel sequencing (454-pyrosequencing). The bioreactor was a two-phase leach-bed process, with separate compartments for cellulolysis and methanogenesis on fixed-films supported by plastic carriers (Bioflow-40 media). 16S rRNA gene sequences and analysis of Pfam protein families were used to describe the structure of both the cellulolytic and methanogenic communities. Archaea represented 2% of the 16S rRNA sequences retrieved from the cellulolytic biofilm and a 12% of the methanogenic biofilm. *Methanomicrobia* dominated in the cellulolytic biofilm (2%), while both *Methanomicrobia* (7%) and *Methanobacteria* (4%) prevailed in the methanogenic biofilm, where the two most abundant genera detected were *Methanosarcina* and *Methanobacterium* (both 4%). The functional analysis supported the evidence of a clear spatial distribution of Archaea between both compartments. Four percent of the environmental genes belonged to Archaea in the cellulolytic biofilm, while the methanogenic biofilm revealed a higher contribution (22%).

The influence of operation conditions on the diversity of archaeal communities in GS, fixed-bed and expanded-bed reactors has been widely investigated in recent years. Two factors often regarded relevant are OLR and HRT. Several studies have been conducted under varying ORL and HRT in reactors treating municipal and industrial wastewaters of diverse nature and operated at different temperatures [37,39,40,44,56,57]. Analyzing the diversity of the prokaryotic communities by means of different molecular approaches, most of these studies concluded that Archaea were less sensitive than bacteria to changes in ORL and HRT [37,39,44,56]. The archaeal community in GS remained rather stable throughout operation, being mainly composed of members of *Methanobacteriaceae*, *Methanosaetaceae* and *Methanosarcinaceae* (Table 1). In contrast, in the packed-bed biofilm reactors the community was dominated by *Methanobacteriaceae*, *Methanomicrobiaceae* and *Methanosarcinaceae*, whose prevalence shifted along the experiments depending on the changes of both ORL and HRT [56,57].

Temperature is one factor that can affect the structure and dynamics of microbial communities in WWT plants; Archaea are not an exception. Several studies have evaluated the effect of temperature on the methanogenic communities in anaerobic bioreactors, comparing their diversity under thermophilic, mesophilic, or psycrophilic conditions. A pioneering work by Visser *et al*. [38] using immunochemical methods revealed differences in the composition of the methanogenic community after a temperature change from 38 to 55 °C, showing that diversity decreased at higher temperatures and that quantitative changes of the size of several subpopulations took place, including *Methanobrevibacter smithii*, *Methanobrevibacter arboriphilus*, *Methanosarcina thermophila*, *Methanospirillum hungatei*, *Methanobacterium thermoautotrophicum*, and *Methanogenium cariaci*. In contrast, Sekiguchi *et al*. [58] analyzed a clone library representing the archaeal community in granules of two UASB reactors fed synthetic wastewater and operated at 35 and 55 °C, and detected a similar composition of the methanogenic communities, composed mainly of *Methanosaeta concilii*, *Methanosaeta thermophila* and populations closely related to the *Methanobacteriales*. Using more sensitive molecular fingerprinting methods, Khemkhao *et al*. [47] evaluated the adaptation of microbial diversity from mesophilic to thermophilic conditions in five consecutive phases (37, 42, 47, 52 and 57 °C) in a UASB granular reactor treating palm oil mill effluent. The results of their study showed that in all cases the acetoclastic methanogens (*Methanosaeta* and *Methanosarcina*) were the dominant Archaea detected in the granules. Also, these authors reported that the dynamics of the archaeal populations were low at temperatures below 52 °C, while important microbial community shifts, particularly of the *Methanosaeta* species, occurred when temperature rose from 52 to 57 °C [47].

Many efforts have been focused to investigate the effect of low temperatures on archaeal diversity in GS anaerobic bioreactors, since the development of a well-functioning psychrophilic microbial consortium is a key factor to keep their operational stability. The results of several studies comparing parallel experiments in bioreactors operated at both psycrophilic and mesophilic conditions are summarized in Table 2, demonstrating temperature-dependent changes of the methanogenic community structure. A general conclusion is that the relative abundance of *Methanosaeta* spp. decreased at 15 °C, favoring the proliferation of *Methanosarcina* spp., and the dominance of the hydrogenotrophic methanogens, particularly the *Methanomicrobiales*. Other available studies reached similar conclusions regarding the diversity of methanogens in anaerobic GS operated at low temperature [59–61]. Besides, O’Reilly *et al*. [45] concluded that the structure of the archaeal communities was drastically changed from that of the seed sludge under mesophilic conditions, while it remained considerably more stable under psychrophilic conditions.

In contrast, few studies have been directed to unravel how low temperatures influence methanogenic populations in anaerobic fixed-bed biofilm systems. 16S rRNA clone libraries and qPCR analyses demonstrated that *Methanomicrobiales* became enriched and displaced the *Methanobacteriales* in a packed-bed biofilm anaerobic reactor when temperature dropped from 18 to 5 °C, while the *Methanosaetaceae* remained at similar levels of abundance throughout the experiment [62]. Members of the *Methanomicrobiales* and *Methanosaetaceae* were able to proliferate and become stably adhered to the carbon fiber carrier; hence, the authors concluded that these archaeal groups had an important role for the efficiency of methanogenesis at low temperatures in this type of system.

The effect of the dissolved oxygen concentration (DO) on the archaeal populations in GS has been also evaluated. Hirisawa *et al*. [64] demonstrated that oxygen concentration did not affect significantly the performance or microbial diversity of UASB granular reactors when operated at different chemical oxygen demand (COD) to sulphate ratios (COD: SO_4_^2−^). Archaea were the dominant domain inside the UASB reactor (68% of the cells) with a DO of 3.0 ± 0.7 mg/L. Under these operational conditions, *Methanosaeta*-like cells were the main methanogens detected by FISH and DGGE fingerprinting. The authors postulated the formation of consortia by the methanogens and facultative bacteria which were able to fast uptake the available O_2_, providing a mechanism of aerotolerance to the methanogens. Additionally, these results were obtained in granular sludge with a large size (2–3 mm diameter), which was found to be the majority of granular biomass inside the reactor (76%). The thickness of the granules and their concentric layered structure acted as a physical barrier to oxygen diffusion, and segregated niches of low-oxygen concentration were generated in their inner zones. Granular sludge of smaller diameter may thus yield different results. The tolerance of methanogenesis to oxygen in GS is of great interest, as this study demonstrated that the application of limited oxygen quantities did not inhibit methanogenesis or sulphate reduction in the UASB, while it allowed a low production of hydrogen sulphide, which is a toxic compound for the hydrogenotrophic methanogens.

The chemical composition of the treated water also affects the characteristics of the microbial communities in GS. Kobayashi *et al*. [65] reported that granulation was enhanced by the addition of certain concentrations of starch-containing waste to an UASB bioreactor treating methanol wastes, increasing the size of the granules formed in the bioreactor. Moreover, the addition of starch led to drastic changes of the structure of the archaeal populations, as revealed by DGGE and FISH. The authors observed that in the absence of starch the main archaeal species in the granular bioreactor were *Methanomethylovorans hollandica*, *Methanobacterium aarhusense*, *Methanobacterium subterraneum* and *Methanolinea tarda*. When starch was added to the UASB, methylotrophic *Methanomethylovorans hollandica* were still the most abundant methanogens, but an important shift of the rest of populations occurred, and *Methanosaeta* spp. (*M. concilii* and *M. barkeri*) became prevalent in the community. The authors pointed to the generation of acetate due to the degradation of the starch by fermentative bacteria as a possible factor influencing the proliferation of the acetoclastic *Methanosaeta* species.

Besides the metabolic role of methanogenic Archaea in anaerobic digestion, their contribution to the stability of GS has been widely reported. Many of the above-mentioned studies highlight that *Methanosaeta* spp. populations are abundant in stable, big-size granules, concluding that these organisms are required for the good performance of anaerobic bioreactors. Due to their filamentous-like morphology, these methanogens have been suggested to act as a backbone for granule initiation, becoming the basis for gathering other granule-forming microorganisms [66,67]. In particular, *Methanosaeta concilii* is believed to play a key role in setting up granulation [68–70]. The initiation of granules by filamentous cells is followed by the subsequent colonization of acetogenic bacteria and hydrogenotrophic methanogens, leading to the layered granular biofilm structure [26].

The diversity or possible roles of Archaea in aerobic granular formation are completely unknown. Recent studies based on molecular tools have thoroughly analyzed the roles of bacteria, ciliated protozoa and fungi on the structuration of granules and their stability; however, the archaeal division was not explored [22,71]. Methanogenic Archaea seem not to be restricted to colonize and form biofilms in engineered systems operated under anaerobic conditions. Goméz-Silván *et al*. [16] analyzed the structure of the archaeal communities in samples of different pilot-scale bioreactors treating wastewater under aerobic conditions, including biofilm samples from submerged fixed-biofilters consisting of one aerated and one anoxic column, and using clayey schists as the biofilm support media. Temperature gradient gel electrophoresis (TGGE) of 16S rRNA gene fragments and phylogenetic analysis of the reamplified TGGE bands demonstrated that populations affiliated to the methanogenic Archaea (*Methanosarcinales*, *Methanobacteriales* and *Methanomicrobiales*) were present in all of the analyzed samples regardless of the aeration conditions, although the composition of the community varied depending of the characteristics of the treated water and the type of technology used. These authors also suggested that the methanogens found in the aerated WWT plants investigated in their study may simply survive under oxygen exposure and be restricted in their activity to the anoxic areas of the plants, or just play structural roles in cell-aggregate development as they are proposed to do in GS. Archaea have also been detected in other aerated WWT systems [72–76].

## 4. Ammonia-Oxidizing Archaea (AOA) in WWT Plants: Occurrence and Significance

Since the first description of an aerobic *Crenarchaeota* group as potential ammonia-oxidizing organisms [77], the global *N*-cycle has been reconsidered. After isolation of the first AOA, *Nitrosopumilus maritimus* [78], only one other isolate—*Candidatus* Nitrososphaera viennensis—has been obtained so far [79], although many other AOA have been enriched from different environments (Table 3). In recent years, a vast number of studies based on molecular tools were performed in natural ecosystems such as soils, oceans or geothermal habitats and allowed the evaluation of the contribution of AOA to ammonia oxidation. In many cases, AOA were found to be dominant over ammonia-oxidizing bacteria (AOB), which were, until then, the only known organisms responsible for the limiting step of nitrification: ammonia oxidation [4,80]. However, with few exceptions [11] the vast majority of studies performed to date have been based on the detection of the archaeal *amoA* gene without demonstrating active ammonia oxidation by AOA, and the presence or high abundance of a functional gene does not mean that its associated function is actually operating. For this reason, some authors proposed the term *amoA*-encoding archaeon (AEA) to refer to these prokaryotic organisms [81–83]. Recently, phylogenetic studies lead to the reclassification of the AOA and AEA as members of a new archaeal Phylum, the *Thaumarchaeota* [4,84,85], and highlighted this group as the potential ancestor of Archaea [85].

Considering that ammonia is the main *N*-species in urban wastewaters, its average concentration, and the fact that the application of the CAS technology under aerated conditions is the most widespread WWT, an important role of AOA in the *N*-removal from the water bodies in engineered systems was initially expected. Using clone libraries, Park *et al*. [95] detected for the first time the presence of AEA in five out of nine different CAS-based WWT plants. However, since then, few studies have proven the presence of AEA in WWT based on different kind of technologies. Many of the available studies also compared the abundance of AEA with that of AOB (Table 4). The results obtained have led to controversial conclusions, reporting either the complete absence of AEA [96], a minimal contribution of AEA to the ammonia-oxidizing community [97–100], an equal contribution [101], or even AEA outcompeting AOB under certain conditions [96,102–104].

AOB and AOA are phylogenetically distant, displaying significant differences in cell physiology and structure, and also demonstrating a significant level of ecological differentiation, as they are present in diverse niches [107,108]. For example, AEA appear to be more sensitive to drought, lysis, temperature and pH changes compared to AOB [107,108]. In the early studies conducted in WWT plants, Park *et al*. [95] pointed out that the AEA occurred in systems with alternation of aeration conditions, with low DO and long retention times (solids retention time, SRT > 15 days, and HRT > 24 h), outcompeting AOB in a system with very low DO (<0.2 mg/L). The kinetic parameter (K_s_) for oxygen described for AOA is slightly lower than the values described for AOB [83], but studies in other WWT systems also detected a high abundance of AEA at higher DO, suggesting that AEA tolerate a wide range of oxygen concentrations [105].

The effect of operational parameters like SRT and HRT is also not clarified. It is well known that longer retention times favor the development of slow-growth microorganisms, as it is the case for both AOB and AOA. In this sense, the membrane bioreactor (MBR) technologies intensify this effect, due to the separation of solids by a filtration process [109]. However, the few studies conducted in WWT plants using this kind of technology did not clarify the positive effect of SRT over the abundance of the AEA community [98,106].

Recent studies described that the most important factor affecting AEA abundance in WWT plants is the available concentration of ammonia. Strong negative correlations are reported among ammonia levels in influent or effluent water and the abundance of archaeal *amoA* copies [104,105,110]. The *K*_s_ for ammonia of AOA is much lower than the values measured for AOB in WWT plants, but the growth rates of AOA are in range with those of the *Nitrosospira*/*Nitrosomonas oligotropha* cluster, with the AOB displaying the higher affinity for ammonia. These data suggest that the AOA are dominant under ammonia-limiting concentrations, whereas these AOB are not able to grow [83]. With ammonia levels closer to their *K*_s_, *Nitrosospira*/*Nitrosomonas oligotropha* cluster and AEA co-dominate, while at higher ammonia concentrations, the AEA seem to be inhibited [83,105]. In general, AOB tend to dominate in systems receiving high direct additions of inorganic ammonia, whereas systems sustained by the mineralization of organic material (ammonification) select for AEA [4]. However, AEA have been recently detected in a CAS system with high influent ammonia concentration [100]. The flocs’ stratification could explain the detection of a sensitive microorganism under suboptimal conditions, but further analyses are required [83].

## 5. Archaea in Biofilms Formed in Membrane Bioreactors (MBR) and their Roles in Biofouling

MBR are an advanced technology that combines the classical biological treatment of wastewater with the use of micro- or ultrafiltration membranes to perform the liquid-solid separation, avoiding the use of the secondary clarifiers [111]. After some decades of existence, membrane bioreactors (MBR) are currently well established as WWT systems which directly compete with the CAS processes due to their many advantages, mostly the generation of pathogen-free treated water that can be directly reused [112]. Compared to CAS, MBR are characterized by a high SRT, which influences the biology of the system, lowering the microbial metabolic activity and growth rates due to the limitation of substrates [113], and favoring the development of slow-growing microorganisms [109]. In both CAS- and MBR-based WWT systems, different populations of microorganism grow together in cell aggregates (flocs), which are stratified structures that are less dense than granules but also hold different microhabitats along their depth [74,75,114].

Anaerobic Membrane Bioreactors (AnMBR) combine an anaerobic bioreactor with a membrane technology for advanced wastewater treatment. There are two main biological focuses of interest in terms of biofilms: the sludge bed of the bioreactor (typically an UASB) where the microbiota is attached to the sludge granules and treats the wastewater, and the biofilm formation on the coupled-membrane surfaces. Hence, the quality of the biofilms supported by the sludge particles and the intimacy of the sludge-wastewater contact are the factors which determine the success of treatment.

The bacterial diversity of MBR is well described; however, the archaeal community remains less explored, with most of the studies being focused on the methanogenic community in AnMBR and, most recently, to AEA in aerobic systems [98,102,110]. Despite their strictly anaerobic metabolism, it has been found that methanogenic Archaea are often part of the microbiota of aerated WWT systems [72–76], and a few studies have also reported their presence in aerated MBR [16,102]. The presence of anaerobic Archaea under aerated conditions is explained by the anoxic microenvironments created by the flocs’ stratification, located in the core of the aggregates. In early studies [75], methanogenic Archaea were detected in activated sludge flocs, but it was not until a few years later that their ability to grow in aerated WWT plants was confirmed [74]. These studies also demonstrated the inactivation of the methanogenesis when the Archaea came into contact with oxygen, but showed that Archaea remained viable and rapidly became active when the anoxic conditions returned. In this sense, the methanogenic Archaea have been described as highly persistent under unfavorable nutritional conditions and tolerant to O_2_ [74,115].

### 5.1. Biofouling in MBR Systems

During the last decades, the interest for the application of the membrane technologies has emerged in WWT. However, one of the drawbacks limiting the use of these systems is biofouling, or the progressive accumulation of pore-blocking materials on the surface of the membranes, due to the growth of microbial biofilms and the subsequent gathering of different types of organic and inorganic materials [112]. The reduction of the permeate efflux and an increase in transmembrane pressure are the major signs of biofouling [116,117]. Consequently, higher energy use and an increase of the frequency of the required chemical cleaning operations of the membranes are needed, which means shorter membrane lifespans and membrane-replacement costs [9]. Hence, better understanding of membrane fouling is not only the key to solving the problem, but is also one of the main factors driving membrane technology forward.

Biofouling starts with the accumulation of microorganisms at the liquid-solid phase transition, occurring by the deposition, growth and metabolism of bacterial cells or flocs on the membranes [118]. Biofilms may or may not uniformly cover the substratum and minimally consist of one or more usually multiple layers of living and dead microorganisms and their associated extracellular products [17,118].

In MBR systems assisted by microfiltration (MF) or ultrafiltration (UF), membrane fouling is a major issue. Although there are various factors that affect membrane fouling on MBR, such as membrane and biomass properties, feed water characteristics and operating conditions, membrane biofouling via microbial products plays a critical role in determining the feasibility of utilizing MBR when compared with other biological processes. Organic colloids and soluble polysaccharides (a part of the bacterial EPS) were found to be the main contributors to membrane fouling and influence the membrane performance in wastewater filtration applications. Studies by Rosenberger *et al*. [119] demonstrated the involvement of fouling in the soluble and colloidal substances in effluents and in the water phase of activated sludge of MBR systems. Bound EPS has been noticed as a key foulant in these systems. Ramesh *et al*. [120,121] fractionated bound EPS into tightly-bound EPS and loosely-bound EPS. They stated that the tightly-bound EPS have the highest fouling potential, while the loosely-bound EPS contribute most of the filtration resistance of the sludge in the MBR.

Recent research has been dedicated to the study of biofouling under a multidisciplinary approach, although these efforts have been mostly focused on membrane technologies applied in aerobic WWT. Many of the available studies aimed for the characterization of the microbial populations responsible for biofouling in MBR and other membrane-based systems, but these have been mainly centered on Bacteria [122–124], and little work is available which has analyzed the relevance of Archaea in biofouling. A recent study by Calderón *et al*. [17] examined the biodiversity of prokaryotic organisms in the fouling biofilms of an AnMBR, based on the UASB technology and coupled to UF membrane modules. They showed that chemical cleaning (NaClO) did not completely remove membrane biofouling, and the populations which remained attached after this operation supported the re-growth of the biofilm, leading to the regeneration of a community of similar structure. 16S rRNA-gene TGGE fingerprints targeting Archaea and sequencing of isolated TGGE bands revealed that the prevalent populations in the foulant layers were closely related to the *Methanospirillaceae* (63% of identified sequences), followed by populations related to *Methanosaeta* spp. Together with methanogenic Archaea, some bacterial populations phylogenetically close to the genus *Sphingomonas* spp. were detected as persistent components of the biofouling. Other authors have also pointed out the involvement of *Sphingomonas* spp. on biofilm formation in membrane systems. Miura *et al*. [122] analyzed for over three months the adhesion and formation of biofilms on the hollow-fiber MF membrane surfaces of a full-scale submerged MBR using real municipal wastewater delivered from the primary sedimentation basin of a municipal WWT facility. The characteristics of the fouling layers were monitored using scanning electron microscopy (SEM), and the composition of planktonic and biofilm microbial communities in the MBR were analyzed using culture-independent molecular-based methods (FISH, 16S rRNA gene clone libraries and phylogenetic analysis), concluding that sphingomonads had an important role in biofouling. These findings are consistent with the well-known ability of sphingomonads to colonize solid surfaces favored by their swarming and twitching motility, where they usually adhere strongly regardless of the surface nature, aided by the production of abundant exopolymers.

As the efficiency of backflushing and NaClO treatment as routine antifouling methods was proven to be limited, the use of alternative strategies was suggested, particularly those specifically directed towards microbial groups shown to be resistant to standard chemical cleaning methods (*i.e*., *Sphingomonadaceae* bacteria and methanogenic Archaea). Overall, the development of more appropriate strategies to control membrane biofouling requires a more thorough understanding of biofilm properties and behavior, especially the early steps in biofilm formation [9]. Currently, control measures for membrane biofouling include applying intermittent suction, improving module configurations, improving aeration, reducing the concentration of suspended solids in the bioreactor, applying a tangential surface shear force, backwashing the membrane module, and adding exogenous antibacterial agents [120,125]. In biological terms, quorum quenching has been developed as a new and prosperous strategy in antifouling [126].

## 6. Future Prospects

The knowledge of archaeal diversity, abundance and functions has considerably increased in the last decades. In particular, their unique role as methanogenic organisms has been a central subject of investigation, and their significance in many ecological niches is currently well understood. Regarding the importance of these organisms in WWT, the structure and dynamics of archaeal communities in granular systems are thoroughly investigated, and the information on the influence of operating conditions on their diversity and performance is extensive. However, analogous research focused on fixed-film and expanded-bed reactors is limited in comparison, even though the benefits of providing a support material for biofilm formation are well acknowledged to improve methanogenesis and the general performance of anaerobic bioreactors. The reasons for the widespread presence of methanogenic Archaea in aerobic WWT, the understanding of their survival strategies in a theoretically hostile environment, the roles they may fulfill in organic matter degradation under aerobic conditions, or their suggested contribution to structural stability of suspended cell aggregates and biofilms, are also challenges for future research on the subject.

The wide distribution of AEA in the environment is well recognized at present. There is ample confirmation of their prevalence over AOB in habitats such as oceans, sprigs, soils or estuarine sediments [11,107,127–129]. However, the abundance of AEA in engineered habitats is reported to be highly variable, and the reasons determining this random distribution remain obscure. In particular, the influence of geography on AEA occurrence is striking. To the best of the authors’ knowledge, there are still no reports of AEA detection in urban WWT plants based in Europe [96], while their presence in WWT systems examined in America and Asia is frequently reported [83]. The adaptation of AOA to low-ammonia levels is suggested by several studies, but other factors such as low carbon substrate availability, low pH, low DO concentration and sulphide content characterize niches where AEA are reported abundant [129]. The survey of AEA occurrence in WWT is still fairly limited, and the information gathered to date is often contradictory; thus, the factors determining the occurrence and abundance of AEA need to be further addressed.

The real contribution of AEA to ammonia oxidation in engineered habitats also needs to be assessed. Mußmann *et al*. [96] found that AEA outnumbered AOB up to 10-fold in a WWT plant treating refinery wastewater, but the application of a nitrification mathematical model, the detection of poor archaeal assimilation of labeled ^13^CO_2_, and FISH-microautoradiography (FISH-MAR) studies performed with ^14^C-inorganic carbon strongly evidenced that AEA were not acting as true chemolithoautotrophic ammonia-oxidizing microbes despite carrying and transcribing the *amoA* gene. The authors failed to find the possible source of carbon used by the AEA, even though they made a great effort applying FISH-MAR using a variety of radiolabeled substrates (amino acids, pyruvate, acetate, benzoate, and phenol). Further research is thus required to reveal the roles and importance of these organisms when expressing a heterotrophic mode of living in WWT, as well as the nature of the substrates that support their growth.

The use of MBR in wastewater treatment is steadily growing due to their many advantages over the CAS process; however, biofouling is a major issue restraining the broad application of this technology. Consequently, control of biofouling has become the main topic in MBR research. Conventional methods applied to minimize or eliminate biofouling often fail, because particular members of the biofilm community are intrinsically resistant to such chemical and physical treatments [17,130]. Alternative antifouling strategies are thus welcomed to efficiently eliminate the persistent components of the fouling biofilms. Methanogenic Archaea have been detected as recalcitrant components of the biofilms fouling membranes in MBR systems [17]. Studies analyzing *de novo* biofilm development inside an UASB reactor conclude that Archaea are absent during the initial phases of biofilm formation, but proliferate during the consolidation stage [131]. The reasons why Archaea are particularly persistent to antifouling strategies remain to be clarified. The unique characteristics of the archaeal cell envelope [132] may contribute to the persistence of these organisms on membrane surfaces.

Information about the mechanisms which control biofilm formation by Archaea is scattered. An endopolysaccharidase (disaggregatase) was isolated from a strain of *Methanosarcina mazei*, which efficiently dispersed the aggregates of *M. mazei* cells and was only secreted at certain stages of their life cycle [133]. The gene encoding the enzyme has been isolated and characterized [134]; however, the regulation of its expression and the possible role of disaggregatase under the biofilm life style have not been yet clarified. The roles of transcriptional regulators of the Lrs14 family in surface attachment and biofilm development have been just recently described in the *Crenarchaeota* [135]. Future studies should bring forth new insights into the regulation of biofilm formation and dispersal in Archaea.

In recent years, several authors have proposed advanced antifouling methods, focused to the particular biological characteristics of the microorganisms that made them able to develop very persistent biofilms. Enzymatic disruption of EPS, addition of chemical uncouplers, quorum-quenchers, or bacterial polysaccharides with antibiofilm activity are some of the methods which have proven effective for the dispersal of bacterial biofilms [136,137]. However, there is virtually no information available on the effectiveness of these approaches on biofilm-forming Archaea, although these prokaryotic organisms have been commonly identified in mixed-population biofilms in both aerated and anaerobic WWT plants.

Inhibition of quorum sensing (QS) by quorum quenchers is one of the more promising biological tools recently introduced to control microbial attachment and membrane fouling [126,137]. QS mechanisms in Archaea are still poorly known. However, the implication of acyl-homoserine lactones (AHLs) as QS signals in methanogenic Archaea has been recently revealed [62]. The *luxI* and *luxR* homologues, *filI* and *filR*, were located in the genome of a *Methanosaeta harundinacea* strain and were confirmed as the determinants of the production of long-chain (C_10_–C_14_) AHLs. The *filIR* genes actively regulate cell assembly by determining the morphology change of *M. harundinacea* from short cells to long filaments, hence controlling the role of these organisms in cell aggregation. The production of AHL-like compounds has been observed in pure cultures of *Methanosarcina mazei* and *Methanothermobacter thermoautotrophicus*, and orthologues of the *filI-filR* genes were also detected in the genomes of several methanogens (*Methanosaeta concilii*, *Methanosaeta thermophila*, *M. mazei* and *Methanospirillum hungatei*). These data suggest that QS mediated by AHLs is widespread in this archaeal clade. Consequently, methanogenic AHLs are promising tools for the promotion of granulation of sludge; at the same time, knowledge of the QS mechanism of these organisms provides new targets for the control of archaeal-related biofouling by means of quorum-quenching.

## Figures and Tables

**Table 1:** Effect of Organic Loading Rate (OLR) and Hydraulic Retention Time (HRT) on the diversity of methanogenic Archaea in anaerobic bioreactors.

Reference	[37]	[39]	[44]	[56]	[57]
Type of bioreactor	EGSB	UASB	EGSB	Packed-bed biofilm	
Nature of wastewater	Leachate from municipal sewage sludge incineration plant	Unbleached cellulose pulp	Oleic acid	Short-chain fatty acids	
Temperature (°C)	33 ± 1	30 ± 3	37	55	
ORL (kg COD/m^3^/day)	3.0 to 18.4	0.53 to 1.40	2 to 8	10 to 129	2.9 to 12.2
HRT (h)	2.5 to 4.0	36 to 24	24	24 to 1.4	15 to 3.6
Method of study of prokaryotic diversity	DGGE, qPCR	SEM, DGGE	DGGE, FISH	Clone library	DGGE
Prevalent Archaea detected	*Methanosaeta* (68.4%) shifting to *Methanosarcina* (62.3%) at the end of the experiment	*Methanosarcina* *Methanosaeta*	*Methanobacterium* *Methanosaeta*	*Methanoculleus* *Methanothermobacter* *Methanosarcina*	

COD: chemical oxygen demand; SEM: scanning electron microscopy; DGGE: denaturing gradient gel electrophoresis; FISH: fluorescence *in situ* hybridization; qPCR: quantitative real-time PCR.

**Table 2:** Effect of temperature on the diversity of methanogenic Archaea in anaerobic bioreactors operated under psycrophilic or mesophilic conditions. See Table 1 footnote for abbreviations.

Reference	[45]	[49]	[63]	
Type of bioreactor	EGSB	EGSB	EGSB	
Nature of wastewater	Synthetic glucose wastewater	Synthetic brewery wastewater	Synthetic wastewater	Synthetic wastewater added with trichloroethylene (10–60 mg/L)
Temperature (°C)	15 and 37	15 and 20	15 and 37	
ORL (kg COD/m^3^/day)	5.8	-	3	
HRT (h)	12	18	24	
Method of study of prokaryotic diversity	DGGE, qPCR	Clone library, DGGE	qPCR	
Archaea detected at both temperatures	*Methanobacterium beijingense* *Methanosaeta concilii*	*Methanobacterium* *Methanosaeta*	*Methanobacteriales* *Methanosaetaceae*	
Archaea favored by psycrophilic conditions	*Methanocorpusculum* *Methanosarcinaceae*	*Methanospirillum* *Methanosphaerula* *Methanometylovorans* *Methanosarcina*	*Methanomicrobiales*	
Archaea favored by mesophilic conditions	*Methanospirillum hungatei*	-	-	
Relevant effects of temperature	qPCR demonstrated important shifts of *Methanosaeta* abundance at 15 °C Hydrogenotrophic methanogens prevailed at 15 °C, particularly *Methanomicrobiales*	Lower temperature decreased the abundance of *Methanosaeta* and led to a higher diversity of methanogens	Start up was slower at 15 °C *Methanomicrobiales* emerged earlier at 15 °C *Methanosaetaceae* response to trichloroethylene toxicity differed with temperature	

**Table 3:** Current status of proposed classification of ammonia-oxidizing *Thaumarchaeota*. Please note that not all the taxonomic names are published validly.

Orders	Genera	Species	Origin	Reference
*Nitrosopumilales* (Group I.1a, marine)	*Nitrosopumilus*	*N. maritimus*	Aquarium in Seattle (USA)	[78]
		*Candidatus* N. koreensis	78-m-deep marine sediment off Svalbard (Arctic Circle)	[86]
		*Candidatus* N. salaria	Sediments in the San Francisco Bay estuary (USA)	[87]
		*Candidatus* N. sediminis	Marine sediment off Svalbard (Arctic Circle)	[88]
	*Candidatus* Nitrosoarchaeum	*Candidatus* N. koreensis	Soil sample from the rhizosphere of *Caragana sinica*	[89]
		*Candidatus* N. limnia	Low-salinity sediments in San Francisco Bay (USA)	[90]
*Cenarchaeales* (Group I.1a associated)	*Cenarchaeum*	*C. symbiosum*	Marine sponge	[91]
	*Candidatus* Nitrosotalea	*Candidatus* N. devanaterra	Acidic soil (pH 4.5)	[92]
*Nitrososphaerales* (Group I.1b, soil)	*Candidatus* Nitrososphaera	*Candidatus* N. viennensis	Garden soil in Vienna (Austria)	[79]
		*Candidatus* N. gargensis	Microbial mats of the Siberian Garga hot spring	[93]
Unclassified *Thaumarchaeota* (Group ThAOA)	*Candidatus* Nitrosocaldus	*Candidatus* N. yellowstoni	Sediment from hydrothermal spring in Yellowstone (USA)	[94]

**Table 4:** Occurrence and abundance of amoA-encoding archaeon (AEA) and ammonia-oxidizing bacteria (AOB) in wastewater treatment (WWT) plants.

Reference	[95]		[99]	[105]		[98]	[106]	[102]	
Method of study	Clone library		qPCR	qPCR		qPCR	Clone library	qPCR	
No. and type of WWT plants	5 AS	4 AS	1 AS	4 urban AS	3 industrial AS	MBR	MBR	3 urban	3 industrial
SRT (days)	17.4	11		17.75	12	Complete retention	15–20		
HRT (h)	40	22.5	6.2	4.5	54.3		8		
COD	540	177	179			465	596	266.3	1334.67
BOD	271.5	254		39.69	984.83	249	333		
Average influent NH_4_^+^ (mg/L)	28.54	24.47	18.9	8.23	180.8	4.8		34.23	121.53
Average effluent NH_4_^+^ (mg/L)	0.16	0.38	0.86	1.2	17.05	0.3	1		
% NH_4_^+^ removal	99.30	97.90	95.45	79.60	83.50	72.00			
DO (mg/L)	3.38	3.80	3.87						
TSS sludge (mg/L)			3335	2815	4177	1,1710	4600		
AEA *	+	−	10^4^–10^6^	10^8^–10^11^	ND (<10^2^)	10^3^–10^4^	+	10^5^–10^6^	10^3^–10^4^
AOB *	+ (except 1)	+	10^8^–10^9^	10^8^–10^10^	10^9^–10^10^	10^5^–10^6^	+	10^3^–10^5^	10^7^–10^9^

AS: activated sludge; MBR: membrane bioreactor; SRT: solids retention time; HRT: hydraulic retention time; COD: chemical oxygen demand; BOD: biological oxygen demand at 5 days; DO: dissolved oxygen; TSS: total suspended solids; ND: not detected.

* clone library: positive (+) or negative (−) detection; qPCR: number of *amoA* gene copies/l activated sludge.
